# Association Between Health-Related Quality of Life and Being an Immigrant Among Adolescents, and the Role of Socioeconomic and Health-Related Difficulties

**DOI:** 10.3390/ijerph110201694

**Published:** 2014-01-30

**Authors:** Michèle Baumann, Kénora Chau, Bernard Kabuth, Nearkasen Chau

**Affiliations:** 1 Integrative Research Unit on Social and Individual Development (INSIDE), Institute Health & Behaviour, University of Luxembourg, Walferdange L-7201, Luxembourg; 2 Service de Pédopsychiatrie, Faculté de Médecine, Université de Lorraine, Hôpital d’Enfants de Nancy-Brabois, Vandoeuvre-lès-Nancy F-54500, France; E-Mails: c.kenora@yahoo.fr (K.C.); bernard.kabuth@yahoo.fr (B.K.); 3 INSERM, U669, Paris F-75014, France; E-Mail: nearkasen.chau@wanadoo.fr; 4 Univ Paris-Sud and Univ Paris Descartes, UMR-S0669, Paris F-75014, France

**Keywords:** immigrant adolescents, health-related quality of life, socioeconomic factors, unhealthy behaviors, violence

## Abstract

To develop satisfactorily, adolescents require good health-related quality of life (QOL, including physical health, psychological health, social relationships and living environment). However, for poorly understood reasons, it is often lacking, especially among immigrants with lower family and socioeconomic resources. This study assessed health-related QOL of European and non-European immigrant adolescents and the contributions of socioeconomic difficulties, unhealthy behaviors, and violence. It included 1,559 middle-school adolescents from north-eastern France (mean age 13.5, SD 1.3; 1,451 French adolescents, 54 European immigrants and 54 non-European immigrants), who completed a self-administered questionnaire including sex, age, socioeconomic characteristics (family structure, parents’ education, occupation, and income), unhealthy behaviors (uses of tobacco/alcohol/cannabis/hard drugs, obesity, and involvement in violence), having sustained violence, sexual abuse, and the four QOL domains measured with the World Health Organization’s WHOQOL-BREF (poor: score < 25th percentile). Data were analyzed using logistic regression models. Poor physical health, psychological health, social relationships, and living environment affected more European immigrants (26% to 35%) and non-European immigrants (43% to 54%) than French adolescents (21% to 26%). European immigrants had a higher risk of poor physical health and living environment (gender-age-adjusted odds ratio 2.00 and 1.88, respectively) while non-European immigrants had a higher risk for all poor physical health, psychological health, social relationships, and living environment (3.41, 2.07, 3.25, and 3.79, respectively). Between 20% and 58% of these risks were explained by socioeconomic difficulties, parts of which overlapped with unhealthy behaviors and violence. The associations between the two sets of covariates greatly differed among French adolescents and immigrants. Poor QOL was more common among European and non-European immigrants due to socioeconomic difficulties and associated unhealthy behaviors and violence. The different risk patterns observed between French adolescents and immigrants may help prevention.

## 1. Introduction

Healthy adolescent development includes self-awareness, positive behaviors, goal setting and achievement, successful transition into adulthood, and good health-related quality of life, trust, optimism, and meaning in life [1,2]. However, early adolescence coincides with the middle-school years, a period of transition from the total social and economic dependence of a child to relative independence with more contacts/exchanges with others and more access to licit and illicit drugs. Research has shown that many adolescents have a high risk for a wide range of health-related difficulties: poor living environment, poor social relationships, unhealthy behaviors (use of tobacco/alcohol/cannabis/hard drugs, obesity, and involvement in violence), poor physical health, poor psychological health, suicidal behaviors, sustained violence (physical/verbal violence and sexual abuse), and involvement in violence [2,3,4,5]. 

With increasing migration, European Union health systems face new challenges (rights to health, access to care, and health monitoring) [6]. Thanks to increasing diversity of populations, schools are now multi-cultural settings, and existing health-related problems can be exacerbated among European immigrants and even more so among non-European immigrants as their families originate from less developed or developing countries with lower gross domestic product per inhabitant [7], and have lower education, socioeconomic status, and resources [8,9,10]. It is thus important to study the adolescent’s perception of his/her position in life in the context of the culture and value systems in which he/she lives and in relation to his/her goals, expectations, standards, and concerns *i.e*., the health-related quality of life (QOL) as defined by The World Health Organization (WHO) [11]. The European Pact signed in 2008 in Brussels recognized that population health and well-being play essential roles in the economic and social success of the Union [12]. The WHO has proposed an appropriate measure, the WHOQOL and, more importantly, a shortened version, the WHOQOL-BREF, in various languages. This measures four important domains: physical health, psychological health, social relationships, and living environment [11]. It has shown to be a good, reliable and valid cross-cultural measure [11] and deserves to be widely used in adolescent studies to evaluate and promote adolescents’ QOL via appropriate preventive measures to address potential causes and covariates. However, although the literature is abundant in adults, it is scarce for young adults and university students [2], and rare for adolescents (most research has focused on patients with specific diseases); available studies have been conducted in Bangladesh, India, Taiwan and Kuwait, not in European and other developed countries [13,14,15,16]. 

A question of interest is whether European and non-European immigrants suffer more than native adolescents from poor QOL (*i.e*., its four dimensions: physical health, psychological health, social relationships, and living environment), and whether potential socioeconomic difficulties (non-intact families, low parents’ education, low father’s occupation level, and insufficient family income) explain the potentially higher risk. Over recent decades, changes have occurred in society and the social environment of adolescents, who now have fewer siblings and more often live with cohabiting, divorced/separated or single parents [17]. Over the last decade, poverty in households with children is rising in nearly all “Organization for Economic Co-operation and Development” (OECD) countries, reaching one in five children in Israel, Mexico, Turkey, the United States and Poland [18]. Because of these socioeconomic difficulties, many adolescents may live in a poor environment, with poor social relationships, and health-related difficulties [1,2,3,4] *i.e*., poor QOL, and the issues are likely to be more pronounced among European immigrants, and more especially among non-European immigrants because of higher socioeconomic difficulties. The possible role of socioeconomic difficulties may be amplified, as physical and psychological disorders among people with social/material deprivation are less likely to be treated [19,20,21,22]. In addition, low socioeconomic status and psychological disorders are associated with a failure to listen and to benefit from treatment explanations by physicians, and with poor adherence to medical treatment [23]. In France, the percentage of people under poverty threshold (<60% of median income) was 7.5% in 2009; it reached 13.7% in individuals aged 18–24, 20.8% in single parent families, and 43.8% in inactive mothers [24]. Four million individuals had no complementary health insurance in 2008 [25]. 

Another question of interest is whether unhealthy behaviors (tobacco/alcohol/cannabis/hard drug use, obesity, and involvement in violence) and sustained violence (physical/verbal violence and sexual abuse) are more common among European and non-European immigrants than among French adolescents, and whether they play a role in poor QOL in addition to socioeconomic difficulties. Indeed, it is well known that unhealthy behaviors and violence are common among adolescents, and, because they are associated with socioeconomic difficulties [1,2,3,4,5], they may be more common among European and non-European immigrants. Tobacco and alcohol consumption affect physical, psychomotor and cognitive performance [26,27,28,29], cannabis use may exacerbate mental health difficulties [30], and early use is associated with a higher risk of suicidal behaviors [3]. Maltreatments such as sexual abuse and physical/verbal violence occur too often [3,31], and are associated with drug use, depressive/internalizing symptoms, child adaptation failure, and damage to cognitive development [31,32,33,34] as well as violent and suicidal behaviors [3,35]. We therefore need to assess the role of unhealthy behaviors and sustained violence in the four dimensions of QOL among European and non-European immigrants and the mediating/moderating roles of socioeconomic difficulties. The results would be expected to reveal the value of the WHOQOL as an indicator of well-being, care need and successful integration of immigrants. Knowledge of risk patterns may be useful for prevention and improve health-related QOL by monitoring health-related difficulties among adolescents at risk. 

In the context of early adolescence in France, this study aimed to assess poor health-related QOL (in its four dimensions of physical health, psychological health, social relationships, and living environment) of European and non-European immigrants and the role of socioeconomic difficulties, unhealthy behaviors, and sustained violence. We focused on students in middle schools, mostly under 16 years, because school is compulsory in France until 16 and many problems (such as health and behavioral difficulties) become more established in late adolescence (16–20 years) and need to be solved sooner. 

## 2. Method

### 2.1. Study Design

The study population comprised all 1,666 students attending three middle schools, two public and one private, chosen as it may reflect a social gradient (various social categories are represented) in the urban area of Nancy (410,000 inhabitants), the capital of the Lorraine region (2,342,000 inhabitants) in north-eastern France. The study population concerned a relatively large geographical area (comprising 38.000 inhabitants) and comprised 63 classes. The investigation was approved by the Nancy-Metz regional education authority and the Commission Nationale de l’Informatique et des Libertés (national review board). Written informed consent was obtained from the respondents. In contrast to national studies in which we have participated [3,4,36,37] this study focused on an exhaustive population from a north-eastern urban area in France, so that the subjects were in the same socioeconomic context, free of regional variations.

The study protocol included an invitation to participate transmitted to parents/guardians (April 2010) and data were collected (May–June 2010) using an anonymous self-administered questionnaire filled in over a one-hour teaching period, under research-team supervision with teacher assistance (for surveillance, with no influence on the survey). Respondents were allowed to ask the two research-team members if they did not understand a question but the team had been instructed not to say anything that might influence the response (they rarely did so). Completed questionnaires were put in sealed envelopes and then a closed box by the subjects. Two students refused and 89 (5.3%) were absent when the data collection was carried out (for reasons independent of the survey). In total, 1,575 subjects (95%) completed the questionnaires; 10 respondents were of unknown gender/age, and six questionnaires were not completed appropriately, leaving 1,559 (94%) available for analysis. This population was close to that of a French school-based population survey in terms of gender, family and health-related factors (Appendix 1).

The questionnaire included demographic and socioeconomic characteristics (age, gender, family structure, parents’ education, father’s occupation, and family income), current alcohol, tobacco, cannabis, hard drug use, involvement in violence, violence sustained by the respondent, sexual abuse, self-reported height and weight, and directly measured height and weight (as in other studies [38]), and the WHOQOL-BREF [11].

### 2.2. Measures

#### 2.2.1. WHOQOL-BREF

The validated French version of WHOQOL-BREF was used [11,39]. In the present study, it had a good internal consistency in its four domains of physical health, psychological health, social relationships, and living environment, with satisfactory Cronbach’s alpha coefficients of 0.72, 0.70, 0.62, and 0.78, respectively [40]. This remained true in French adolescents (0.71, 0.70, 0.62, and 0.78, respectively) as well as in European immigrants (0.77, 0.71, 0.61, and 0.76, respectively) and in non-European immigrants (0.72, 0.74, 0.62, and 0.77, respectively). The four domains were one-dimensional as indicated by factor analyses that yielded first eigenvalues of 2.00, 1.97, 0.66 and 2.64, much higher than the 2nd eigenvalues of 0.26, 0.14, −0.25 and 0.35, respectively. The four domains remained one-dimensional in French adolescents (first eigenvalues of 1.98, 1.96, 0.64 and 2.61, respectively, much higher than the 2nd eigenvalues of 0.27, 0.14, −0.24 and 0.34, respectively) as well as in European immigrants (first eigenvalues were 2.41, 2.02, 0.74 and 2.70, respectively that were much higher than the 2nd eigenvalues of 0.52, 0.31, −0.25 and 0.66, respectively) and in non-European immigrants (first eigenvalues of 2.00, 2.10, 0.65 and 2.87, respectively, much higher than the 2nd eigenvalues of 0.59, 0.29, −0.25 and 0.78, respectively). We used the 25th percentile as a cut-off value (the quartiles are often used for deprivation measures [41]) which appears appropriate for most subjects with health-related issues. The results of the present study may show the usefulness of the WHOQOL-BREF in adolescent surveys.

#### 2.2.2. Father’s Occupational Category and Income

Five categories were considered following the international classification of occupations (ISCO): managers, professionals, and intermediate professionals; craftsmen, tradesmen, and heads of firms; service workers and clerks; manual workers and other occupations; and non-working people (unemployed and retired). For perceived income, subjects were asked whether, financially, their family was: coping but with difficulties/getting into debt *vs*. comfortable/well off/earning just enough [42,43].

#### 2.2.3. Current Alcohol, Tobacco Cannabis, and Hard Drug Use

Use of these substances was assessed with the questions “During the last 30 days”: “how many times have you had alcoholic drinks (beer, cider, champagne, wine, aperitif, *etc*.?” (None/1–5/6–9/10–29/30+), “how many cigarettes a day did you smoke?” (None/1–4/5–9/10–19/20+ cigarettes/day), “on how many occasions have you used any form of cannabis?” (None/1–5/6–9/10–29/30+), and “on how many occasions have you used any form of other illicit drugs (mushrooms, ecstasy, LSD, *etc*.)?” (None/1–5/6–9/10–29/30+) [3,4,36,37]. These factors were dichotomized (at least one *vs*. none).

#### 2.2.4. Violence Sustained by the Respondent

This was measured using a 20-item scale (five questions for four localities: in school, school neighbourhood, at home, and elsewhere) [3,4]: “During the last 12 months, have you been victim of …?”: hitting, stealing, racket, insult, and racial abuse (yes *vs*. no). Cronbach’s alpha was satisfactory (0.71) [40], allowing a single score to be calculated. Violence sustained was defined by the presence of at least one item.

#### 2.2.5. Sexual Abuse

This was probed for with the question: “In the course of your life, have you been a victim of sexual abuse?” (yes *vs*. no) [3,4]. 

#### 2.2.6. Involvement in Violence

This was measured with a 11-item scale [3,4]: “During the last 12 months, have you?”, “gotten mixed into a fight at school”, “taken part in a fight where a group of your friends were against another group”, “belonged to a group starting a fight against another group”, “committed insults”, “committed racial abuse”, “started a fight with another individual”, “taken something not belonging to you (in school, in the neighbourhood of school, at home, ...”, “taken something from a shop without paying for it”, “set fire to somebody else’s property on purpose”, “used any kind of weapon to get something from a person”, or “damaged public or private property on purpose” (yes *vs*. no). Cronbach’s alpha was satisfactory (0.82) [40], allowing a single score to be calculated. Involvement in violence was defined by the presence of at least one item.

#### 2.2.7. Weight and Height Self-reporting and Measurement

Self-reports were obtained from two questions: “About how much do you weigh without clothes and shoes?”, “About how tall are you without shoes?” [44]. During questionnaire completion and after reporting weight and height, all adolescents were invited to measure their weight and height with the same research-team trained physician. Weight and height measurements were performed in a dedicated area and a second research-team member ensured that peers could not come near. The teachers were not allowed to come close either. Thus, no-one else could read the measurements. Body height was measured with a measuring tape (mounted on a portable stadiometer fixed on the wall) and weight with Scaleman electronic scales (accurate to 50 grams). Measurements were taken without shoes in a light gown. The body mass index (BMI) was defined as weight/height^2^ (kg/m^2^) using measured values or, if these are missing, self-reported values. BMI was then categorized into underweight, normal weight, overweight or obese according to the widely used threshold values recommended for male and female French adolescents at different ages [45]. 

### 2.3. Statistical Analysis

The item analysis of the WHOQOL-BREF was performed using mean value, Pearson correlation coefficient, and Cronbach’s alpha coefficient. The difference for each WHOQOL-BREF item/domain between European or non-European immigrants and French adolescents was assessed using multiple linear regression coefficients taking gender and age into account. Then poor physical health, psychological health, social relationships, and living environment were defined using the 25th percentile as a cut-off value. Their relationships with nationality, socioeconomic factors, unhealthy behaviors, having sustained violence and sexual abuse were assessed using the Chi^2^ and Fisher tests and gender-age-adjusted odds ratios (gaOR) and 95% confidence intervals (CI) computed via logistic regression models. The gaOR was considered taking gender and age into account before we examined the role of socioeconomic difficulties and that of unhealthy behaviors, having sustained violence, and being a victim of sexual abuse. Age also reflects the exposure duration for various issues. To study the associations between each outcome variable and ethnic group, three logistic models were performed: a basic model (model 1) measured the associations adjusting for gender and age, and then with further adjustment for socioeconomic factors (model 2), and finally with further adjustment for unhealthy behaviors, having sustained violence, and being victim of sexual abuse (model 3). The contribution of these factors to the explanation of ethnic group-outcome variable association was estimated by the change in the ORs *i.e*., explained fraction calculated by the formula: (OR_model 1_ – OR_model 2_)/(OR_model 1_ – 1) or (OR_model 1_ – OR_model 3_)/(OR_model 1_ – 1) [46]. Positive % values indicate reductions in ORs. The contribution was calculated only if the OR_model 1_ was significant. The analyses were performed using the Stata program (Stata Corporation, College Station, TX, USA, 2007).

## 3. Results

The characteristics of WHOQOL-BREF are shown in Table 1. The Pearson correlation coefficient between each item and its domain was between 0.45 and 0.76 for physical health, between 0.43 and 0.73 for psychological health, 0.85 for social relationships, and between 0.56 and 0.69 for living environment. The score of psychological health had the lowest value (mean 62.8). The other WHOQOL-BREF domains had close mean values (between 75 and 78). Compared with French adolescents, non-European immigrants had much lower value for nearly all WHOQOL-BREF items while European immigrants had lower values for body image, safety, and access to health care only.

Table 2 reveals that significant differences were observed between French adolescents, European immigrants, and non-European immigrants for all WHOQOL-BREF domains, age, family structure, father’s occupation, parents’ education, insufficient family income, the consumption of tobacco, cannabis, and hard drugs, obesity, and being a victim of sexual abuse.

Based on gaOR provided in Table 3, non-European immigrants had higher risk for poor physical health (gaOR 3.41), poor psychological health (2.07), poor social relationships (3.25), and for poor living environment (3.79), compared to French adolescents. European immigrants had higher risk for poor physical health and poor living environment only compared to French adolescents (gaOR 2.00 and 1.88, respectively). Girls had higher risk for poor physical health and poor psychological health than boys. Older age was associated with a higher risk of poor physical health, psychological health, and social relationships. Adolescents living in non-intact families had higher risk for all WHOQOL-BREF domains. Compared to adolescents having a father being a manager, professional or intermediate professional, those having a non-working father were at higher risk for all WHOQOL-BREF domains, while those whose fathers were manual workers had a higher risk of poor physical health, psychological health, and living environment. Insufficient family income was associated with all WHOQOL-BREF domains. Unhealthy behaviors, having sustained violence, and being a victim of sexual abuse were associated with all WHOQOL-BREF domains too except between alcohol and cannabis use and social relationships, and between overweight and social relationships.

Table 4 shows that, for European immigrants, the gaOR for poor physical health (2.00) decreased to 1.66 (non-significant, contribution 34%) and that for poor living environment (1.88) decreased to 1.37 (non-significant, contribution 58%) with further adjustment for socioeconomic factors. For non-European immigrants, socioeconomic factors had a contribution of 44% to the risk of poor psychological health (which became non-significant) and about 20% to the risk of poor physical health, social relationships and living environment. For European as for non-European immigrants the odds ratios changed little with further adjustment for unhealthy behaviors, having sustained violence, and being a victim of sexual abuse. 

Table 5 shows that the low contribution of unhealthy behaviors, having sustained violence, and being victim of sexual abuse concealed different patterns of associations between these issues and socioeconomic factors among French adolescents and immigrants. Indeed, girls had a lower risk for alcohol use, obesity and having sustained violence among French adolescents (gaOR about 0.70) and not among immigrants. Older age was associated with having sustained violence among French adolescents only (1.12 per year). Living in a non-intact family was associated with a higher risk for immigrants than for natives of tobacco use (gaOR 6.22 *vs*. 2.93) and hard drug use (gaOR 9.62 *vs*. 1.90, non-significant) in contrast to involvement in violence (gaOR 1.30, non-significant *vs*. 1.77) and being a victim of sexual abuse (gaOR 1.22, non-significant *vs*. 2.29). Low parents’ education was associated with obesity among French adolescents only (gaOR 1.60). Having a father who was a manual worker or not working played a protective role for alcohol and cannabis use among immigrants (gaOR 0.37 and 0.13, respectively) but not among French adolescents. Insufficient family income was associated with hard drug use, obesity, involvement in violence, and having sustained violence among French adolescents only (gaOR between 1.57 and 2.51).

**Table 1:** Characteristics of WHOQOL-BREF and comparison of European immigrants, non-European immigrants with their French counterparts (N = 1559).

	Mean (SD)	Range	Subjects with Minimal Value (%)	Subjects with Maximal Value (%)	Pearson Correlation Coefficient ^a^	European Immigrants ^b^ (Regression Coefficient (SE))	Non-European Immigrants ^b^ (Regression Coefficient (SE))
**Physical health**	*76.3 (15.5)*	*6–100*	*0*	*5.2*		−3.07 (2.08)	**−9.27 ^‡^** (2.08)
Pain	4.43 (0.88)	1–5	1.5	62.2	0.53	0.024 (0.12)	−0.301 * (0.12)
Independence on medical aids	4.60 (0.96)	1–5	3.6	80.8	0.45	−0.124 (0.13)	−0.117 (0.13)
Energy	3.87 (1.16)	1–5	5.2	38.2	0.67	−0.219 (0.16)	**−0.619 ^‡^** (0.16)
Mobility	4.24 (0.87)	1–5	1.3	47.1	0.50	−0.086 (0.12)	**−0.358 ^†^** (0.12)
Sleep	3.49 (1.20)	1–5	9.1	21.0	0.68	−0.040 (0.16)	−0.253 (0.16)
Capacity for daily living activities	4.06 (0.91)	1–5	1.9	35.3	0.76	−0.061 (0.12)	**−0.414 ^‡^** (0.12)
Work capacity	3.64 (1.09)	1–5	5.5	23.2	0.66	−*0.276* (0.15)	**−0.581 ^‡^** (0.15)
**Psychological health**	*62.8 (19.3)*	*0–100*	*0 .2*	*1.3*		−3.09 (2.60)	−*4.79* (2.60)
Positive feeling	3.77 (1.18)	1–5	6.4	33.3	0.73	−0.245 (0.16)	**−0.597 ^‡^** (0.16)
Personal belief	2.24 (1.42)	1–5	46.2	11.6	0.43	0.002 (0.20)	**0.798 ^‡^** (0.20)
Concentration	3.82 (1.17)	1–5	5.4	36.4	0.59	−0.212 (0.16)	−*0.276* (0.16)
Body image	3.57 (1.34)	1–5	9.8	35.1	0.72	−0.348 * (0.18)	**−0.453 ^†^** (0.18)
Self-esteem	3.67 (1.14)	1–5	6.4	27.1	0.73	−0.996 (0.15)	−0.348 * (0.15)
Negative feeling	3.97 (1.06)	1–5	3.7	37.0	0.63	0.184 (0.14)	−*0.239* (0.14)
**Social relationships**	*78.0 (21.1)*	*0–100*	*1.0*	*29.1*		1.40 (2.89)	**−15.26 ^‡^** (2.90)
Personal relationships	4.06 (1.00)	1–5	3.1	39.3	0.85	−0.102 (0.14)	**−0.629 ^‡^** (0.14)
Support from friends	4.17 (0.99)	1–5	3.1	46.7	0.85	0.203 (0.14)	**−0.586 ^‡^** (0.14)
**Living environment**	*75.4 (17.8)*	*0–100*	*0.3*	*9.2*		−5.13 * (2.43)	**−13.71 ^‡^** (2.44)
Safety	3.78 (1.18)	1–5	5.8	33.8	0.68	−0.073 (0.16)	**−0.486 ^†^** (0.16)
Healthy physical environment	3.43 (1.31)	1–5	11.7	26.2	0.56	−0.097 (0.18)	**−0.468 ^†^** (0.18)
Financial resources	3.58 (1.33)	1–5	10.5	33.9	0.60	−0.277 (0.18)	−0.275 (0.18)
Acquiring information	3.68 (1.20)	1-5	7.4	30.5	0.69	−0.351 (0.17)	**−0.749 ^‡^** (0.17)
Leisure activity	4.24 (1.11)	1–5	4.4	58.6	0.66	−*0.282* (0.15)	**−0.571 ^‡^** (0.15)
Home environment	4.31 (0.95)	1–5	2.1	55.7	0.62	−0.016 (0.13)	**−0.804 ^‡^** (0.13)
Access to health care	4.44 (0.84)	1–5	1.5	60.7	0.66	**−0.302 ^†^** (0.12)	**−0.466 ^‡^** (0.12)
Transport	4.17 (0.97)	1–5	2.8	45.7	0.62	−0.135 (0.13)	**−0.526 ^‡^** (0.13)

Notes: * *p* < 0.05, **^†^** *p* < 0.01, **^‡^** *p* < 0.001. All items and the four domains were considered as continuous variables. **^a^** between an item and the score of its domain. **^b^** *versus* French adolescents, via multiple linear regression model taking gender and age into account. Bold types: values significantly different from zero (*p* < 0.05). Italic types: values close to significance (0.05 < *p* < 0.10). The four domains physical health, psychological health, social relationships, and living environment had a good internal consistency with Cronbach’s alpha coefficients of 0.72, 0.70, 0.62, and 0.78, respectively.

**Table 2:** Associations between nationality and various factors: % or mean (SD).

Factor	French	European immigrants	Non-European immigrants	*p*-value ^a^
*Number of subjects*	*1,451*	*54*	*54*	
**WHOQOL-BREF ≤ 25^th^ percentile value**				
Poor physical health	21.7	35.2	50.0	<0.001
Poor psychological health	26.1	35.2	42.6	0.01
Poor social relationships	25.6	25.9	53.7	<0.001
Poor living environment	23.6	37.0	53.7	<0.001
**Socioeconomic Factors**				
Girls	50.0	55.6	48.2	NS
Age (year)				
Mean age (SD)	13.5 (1.2)	13.3 (1.1)	13.9 (1.7)	<0.05
Range	9.9–16.9	11.7–15.7	11.1–18.7	
Family structure				<0.001
Intact	63.8	57.4	46.3	
Parents divorced/separated and reconstructed family	24.7	35.2	25.9	
Single parent and other situations	11.5	7.4	27.8	
Low parents’ education (<university)	47.6	66.7	59.3	<0.01
Father’s occupation				<0.001
Manager, professional, and intermediate professional	39.5	22.2	18.5	
Craftsman, tradesman, and head of firm	19.9	24.1	22.2	
Service worker and clerk	9.2	5.6	13.0	
Manual worker and other occupations	24.5	38.9	24.1	
Non-working	6.9	9.3	22.2	
Insufficient family income	16.9	25.9	31.5	<0.01
**Unhealthy Behaviors and Violence**				
Substance use				
Tobacco	10.5	16.7	24.1	<0.01
Alcohol	35.6	31.5	29.6	NS
Cannabis	5.1	9.3	14.8	<0.01
Hard drugs	2.3	7.4	11.1	<0.001
Body mass index				<0.001
Underweight	1.4	0.0	1.9	
Normal weight	59.3	42.6	44.4	
Overweight	26.6	42.6	40.7	
Obese	11.5	7.4	13.0	
Missing value	1.2	7.4	0.0	NS
Involvement in violence	59.1	63.0	66.7	NS
Having sustained violence	53.3	55.6	53.7	NS
Being victim of sexual abuse	3.4	1.8	11.1	<0.01

Notes: **^a^** Chi^2^ test or Fisher’s test; NS: non-significant (*p* > 0.05).

**Table 3:** Factors associated with physical health, psychological health, social relationships, and living environment: gender-age-adjusted odds ratio and 95% confidence interval.

	WHOQOL-BREF			
	Poor physical health	Poor psychological health	Poor social relationships	Poor living environment
**Nationality**				
French	1.00	1.00	1.00	1.00
European immigrant	2.00 * 1.11–3.58	1.51 0.85–2.70	1.03 0.55–1.91	1.88 * 1.07–3.31
Non-European immigrant	3.41 ^‡^ 1.94–6.00	2.07 * 1.18–3.63	3.25 ^‡^ 1.88–5.64	3.79 ^‡^ 2.18–6.57
**Socioeconomic Factors**				
Girls	1.70 ^‡^ 1.33–2.16	1.98 ^‡^ 1.57–2.49	0.99 0.79–1.24	1.22 0.97–1.54
Age (per year)	1.28 ^‡^ 1.17–1.41	1.12 * 1.02–1.22	1.10 * 1.00–1.20	1.01 0.92–1.10
Family structure				
Intact	1.00	1.00	1.00	1.00
Parents divorced/separated and reconstructed family	2.13 ^‡^ 1.62–2.79	2.05 ^‡^ 1.58–2.66	1.44 ^†^ 1.11–1.86	2.20 ^‡^ 1.70–2.86
Single parent and other situations	2.06 ^‡^ 1.44–2.93	2.38 ^‡^ 1.70–3.33	1.55 ^†^ 1.11–2.18	2.16 ^‡^ 1.53–3.04
Low parents’ education (<university)	1.83 ^‡^ 1.44–2.34	1.47 ^‡^ 1.17–1.85	1.33 ^†^ 1.06–1.67	2.82 ^‡^ 2.21–3.59
Father’s occupation				
Manager, professional, and intermediate professional	1.00	1.00	1.00	1.00
Craftsman, tradesman, and head of firm	1.27 0.90–1.78	1.04 0.75–1.44	1.04 0.76–1.43	1.25 0.88–1.78
Service worker and clerk	0.98 0.61–1.57	1.20 0.79–1.84	0.98 0.64–1.51	1.48 0.95–2.33
Manual worker and other occupations	1.69 ^‡^ 1.25–2.29	1.43 * 1.07–1.91	1.10 0.82–1.48	3.27 ^‡^ 2.43–4.42
Non-working	1.97 ^†^ 1.26–3.07	2.46 ^‡^ 1.62–3.74	2.31 ^‡^ 1.53–3.49	3.87 ^‡^ 2.52–5.94
Insufficient family income	2.53 ^‡^ 1.91–3.36	2.94 ^‡^ 2.24–3.87	2.31 ^‡^ 1.76–3.04	4.64 ^‡^ 3.53–6.12
**Unhealthy Behaviors and Violence**				
Substance use				
Tobacco	3.18 ^‡^ 2.27–4.44	3.46 ^‡^ 2.48–4.84	1.68 ^†^ 1.20–2.36	2.54 ^‡^ 1.82–3.54
Alcohol	1.34 * 1.04–1.73	1.84 ^‡^ 1.44–2.34	1.02 0.80–1.30	1.40 ^†^ 1.10–1.79
Cannabis	1.66 * 1.04–2.66	1.90 ^†^ 1.20–3.01	1.16 0.72–1.87	1.57 0.98–2.51
Hard drugs	2.84 ^‡^ 1.51–5.32	4.59 ^‡^ 2.43–8.68	1.93 * 1.04–3.60	3.33 ^‡^ 1.80–6.15
Body mass index				
Underweight	0.67 0.19–2.31	1.33 0.50–3.53	1.02 0.37–2.83	1.12 0.40–3.09
Normal weight	1.00	1.00	1.00	1.00
Overweight	1.47 ^†^ 1.12–1.93	1.46 ^†^ 1.13–1.90	1.29 0.99–1.67	1.39 * 1.06–1.81
Obese	1.80 ^†^ 1.24–2.61	2.32 ^‡^ 1.64–3.29	1.74 ^†^ 1.23–2.46	2.03 ^‡^ 1.43–2.87
Missing value	1.39 0.52–3.71	2.84 * 1.17–6.90	2.35 0.97–5.65	2.17 0.89–5.33
Involvement in violence	2.27 ^‡^ 1.73–2.97	2.45 ^‡^ 1.89–3.17	1.45 ^†^ 1.14–1.85	2.51 ^†^ 1.93–3.27
Having sustained violence	1.67 ^‡^ 1.31–2.14	1.75 ^‡^ 1.38–2.21	1.82 ^‡^ 1.44–2.29	1.88 ^‡^ 1.48–2.38
Being victim of sexual abuse	6.60 ^‡^ 3.71–11.8	7.11 ^‡^ 3.91–12.9	3.91 ^‡^ 2.27–6.71	5.47 ^‡^ 3.15–9.53

Notes: * *p* < 0.05; **^†^** *p* < 0.01; **^‡^** *p* < 0.001.

**Table 4:** Associations of WHOQOL-BREF and nationality and role of socioeconomic, unhealthy behaviors and violence: odds ratio (OR), 95% confidence interval (CI), and contribution of covariates (%) **^a^**.

	WHOQOL-BREF			
	Poor physical health OR 95% CI %	Poor psychological health OR 95% CI %	Poor social relationships OR 95% CI %	Poor living environment OR 95% CI %
Odds ratio (OR_1_) adjusted for gender and age				
French	1.00	1.00	1.00	1.00
European immigrants	2.00 * 1.11–3.58 100	1.51 0.85–2.70 ‒	1.03 0.55–1.91 ‒	1.88 * 1.07–3.31 100
Non-European immigrants	3.41 ^‡^ 1.94–6.00 100	2.07 * 1.18–3.63 100	3.25 ^‡^ 1.88–5.64 100	3.79 ^‡^ 2.18–6.57 100
Odds ratio (OR_2_) with further adjustment for socioeconomic factors				
French	1.00	1.00	1.00	1.00
European immigrants	1.66 0.90–3.07 34	1.33 0.72–2.45 ‒	0.91 0.48–1.72 ‒	1.37 0.74–2.57 58
Non-European immigrants	2.93 ^‡^ 1.62–5.31 20	1.60 0.88–2.90 44	2.78 ^‡^ 1.58–4.92 21	3.24 ^‡^ 1.77–5.93 20
Odds ratio (OR_3_) with further adjustment for unhealthy behaviors, having sustained violence, and being a victim of sexual abuse				
French	1.00	1.00	1.00	1.00
European immigrants	1.72 0.91–3.24 28	1.27 0.67–2.43 ‒	0.89 0.46–1.70 ‒	1.32 0.69–2.53 64
Non-European immigrants	2.86 ^‡^ 1.55–5.27 23	1.53 0.82–2.87 50	2.72 ^‡^ 1.53–4.86 24	3.24 ^‡^ 1.75–6.02 20

Notes: * *p* < 0.05, **^†^** *p* < 0.01, **^‡^** *p* < 0.001. Bold type: Significant OR (*p* < 0.05). **^a^** % = Reduction (positive %) or increase (negative %) in OR computed with the following formula: (OR_1_ – OR_2_)/(OR_1_ – 1) or (OR_1_ – OR_3_)/(OR_1_ – 1); calculated for OR_1_ significant only.

**Table 5:** Associations between socioeconomic factors and unhealthy behaviors and violence (N = 1,559): gender-age-adjusted odds ratio and 95% confidence interval.

	N	Tobacco use	Alcohol use	Cannabis use	Hard drug use	Obesity	Involvement in Violence	Having Sustained Violence	Being Victim a of Sexual Abuse
**French Adolescents**									
Girls	725	1.24 0.87–1.74	0.76 * 0.61–0.95	0.64 0.39–1.03	0.64 0.31–1.28	**0.64 ^†^** 0.46–0.89	0.35 ^‡^ 0.28–0.43	**0.66 ^‡^** 0.53–0.81	1.63 0.91–2.92
Age (per year)	1,451	1.58 ^‡^ 1.37–1.82	1.61 ^‡^ 1.47–1.77	1.79 ^‡^ 1.46–2.19	**1.28** 0.97–1.70	0.91 0.80–1.04	1.30 ^‡^ 1.19–1.42	*1.12* ^†^ 1.03–1.22	1.35 ^†^ 1.07–1.69
Family structure									
Intact	926	1.00	1.00	1.00	1.00	1.00	1.00	1.00	1.00
Non-intact	525	**2.93 ^‡^** 2.07–4.17	1.61 ^‡^ 1.28–2.03	2.35 ^‡^ 1.46–3.80	**1.90** 0.95–3.80	1.18 0.84–1.64	*1.77* ^‡^ 1.40–2.23	1.26 * 1.01–1.56	*2.29* ^†^ 1.29–4.05
Low parents’ education	691	1.18 0.84–1.65	0.75 ^†^ 0.59–0.93	1.02 0.63–1.64	1.17 0.59–2.34	*1.60* ^†^ 1.16–2.22	1.18 0.95–1.47	0.97 0.79–1.20	1.68 0.94–2.99
Father’ occupation									
Managers, professionals, and intermediate professionals, craftsmen, tradesmen, and firm heads, service workers and clerks	996	1.00	1.00	1.00	1.00	1.00	1.00	1.00	1.00
Manual workers, other occupations, and non-working	455	1.37 0.97–1.95	*0.84* 0.66–1.07	*0.92* 0.55–1.53	0.78 0.69–3.73	2.29 ^‡^ 1.65–3.19	1.31 * 1.03–1.67	1.18 0.94–1.48	1.75 0.99–3.10
Insufficient family income	245	1.70 ^†^ 1.13–2.54	1.12 0.83–1.50	1.58 0.90–2.76	*2.51* * 1.20–5.25	*1.57* * 1.06–2.33	*1.63* ^†^ 1.20–2.20	*2.35* ^‡^ 1.74–3.16	3.68 ^‡^ 2.05–6.59
**Immigrant Adolescents**									
Girls	56	0.96 0.36–2.53	**1.42** 0.60–3.37	0.58 0.17–2.00	0.71 0.18–2.79	**1.61** 0.44–5.97	0.39 * 0.17–0.93	**0.84** 0.39–1.83	1.06 0.22–5.12
Age (per year)	108	1.47 * 1.05–2.07	1.47 * 1.07–2.00	1.83 ^†^ 1.20–2.78	1.78 * 1.13–2.81	1.13 0.73–1.75	1.40 * 1.01–1.93	*0.88* 0.67–1.16	1.45 0.87–2.39
Family structure									
Intact	56	1.00	1.00	1.00	1.00	1.00	1.00	1.00	1.00
Non-intact	52	**6.22 ^†^** 1.91–20.3	1.51 0.64–3.57	3.46 0.86–14.0	9.62 * 1.15–80.8	0.83 0.23–2.98	*1.30* 0.57–2.98	1.17 0.54–2.53	*1.22* 0.25–6.00
Low parents’ education	68	0.90 0.32–2.53	0.65 0.27–1.60	0.59 0.15–2.32	2.10 0.38–11.6	*0.69* 0.19–2.51	1.18 0.51–2.74	0.84 0.37–1.88	0.32 0.06–1.76
Father’ occupation									
Managers, professionals, and intermediate professionals, craftsmen, tradesmen, and firm heads, service workers and clerks	57	1.00	1.00	1.00	1.00	1.00	1.00	1.00	1.00
Manual workers, other occupations, and non-working	51	0.55 0.20–1.50	*0.37* * 0.15–0.93	*0.13* * 0.03–0.68	0.46 0.11–1.96	1.95 0.52–7.24	1.32 0.57–3.05	0.60 0.27–1.30	1.23 0.25–6.08
Insufficient family income	31	1.60 0.58–4.46	0.82 0.31–2.12	0.72 0.18–2.86	*1.20* 0.29–5.06	*0.79* 0.19–3.34	*1.11* 0.43–2.85	*0.77* 0.33–1.84	2.98 0.59–15.0

Notes: * *p* < 0.05, **^†^** *p* < 0.01, **^‡^** *p* < 0.001. N: number of subjects. Because of small number of subjects, European and non-European immigrants were grouped, and family structure and father’ occupation were dichotomised. In bold types the odds ratios higher for immigrants and in italic types those higher for French (with no consideration of *p*-values which greatly depended on N).

## 4. Discussion

This study is the first to report poor health-related QOL (in its four domains: physical health, psychological health, social relationships, and living environment) among European and non-European immigrants in early adolescence, and the concurrent roles of a wide range of socioeconomic difficulties, unhealthy behaviors, and violence sustained. European immigrants, and especially non-European immigrants, were more subject to poor physical health, psychological health, social relationships, and living environment (gaOR between 1.88 and 3.79), and the risk was largely explained by socioeconomic factors (contributions between 20% and 58%). Unhealthy behaviors and violence sustained contributed little in addition to these factors. However, the associations between these two sets of covariates greatly differed between French adolescents and immigrants. We focused on socioeconomic difficulties, unhealthy behaviors and violence because these issues are common and start in early adolescence [1,2,3,4,5]. The present study also shows the usefulness of the WHOQOL-BREF in assessing health-related QOL, and the role of potential factors in adolescent surveys in a multi-cultural setting. These findings may be important for public policy aiming at reducing health-related difficulties, evaluating care need, and improving integration of immigrants.

First, our results confirm those of the few available studies that the WHOQOL-BREF is valid and can be used in adolescents [13,14,15,16]. We found that its domains were one-dimensional and their internal consistency was similar to those of these studies and others on young adults and university students [15,47,48]. With exchanges and migrations between countries, our society is now more multicultural and the WHOQOL-BREF appeared to be a good tool, easy to use, with which to assess and monitor adolescent health-related problems. In the absence of reference values, Cummins proposed a mean value of 75.0 + 2.5 for general populations [49]. The scores for physical health, social relationships, and living environment found in our study may thus be considered correct. However, the score for psychological health was much lower (62.8), which suggests that adolescents were more affected by this issue than by the other QOL domains. One study on high-school students aged 14–23 in Kuwait reported similar results (61.9 for psychological health and between 70 and 72.8 for other domains) [14]. It is not possible to compare our findings with those of other adolescent studies as they did not investigate socioeconomic factors, unhealthy behaviors and violence but focused more on the validation, reliability and applicability of the WHOQOL-BREF [13,14,15]. Our study is thus an addition to the literature.

In France, European immigrants are from developed countries while most non-European immigrants are from developing countries. Indeed, in 2007, among 2.56 million children aged <18 in immigrant families, the birth country of the family head was in Africa (1.37 million children, mainly in Algeria, Morocco, and Tunisia), Europe (642,302 children, mainly in Portugal, Spain, Italy, and the United Kingdom), Asia (393,775 children, mainly in Turkey and Indochina), and America and Oceania (147,326 children) [50]. It is not easy for non-European immigrants to come in France, unlike European adolescents. Our study shows that European and non-European immigrants were subject to poor quality of life but the magnitude and risk patterns were very different. European immigrants had a higher risk of poor physical health and poor living environment only, and socioeconomic factors partly explained the risks. Non-European immigrants had a much higher risk of poor physical health, psychological health, social relationships, and living environment, and socioeconomic factors explained a great part of the risk for various QOL domains, especially poor psychological health. This may be because socioeconomic features differed a lot between European and non-European immigrants. European immigrants more frequently had divorced/separated parents or reconstructed families and a father being a manual worker, while non-European immigrants more often had single parents and a non-working father; both groups more often had low parental education; and insufficient family income was more common among non-European immigrants. Having divorced/separated parents or reconstructed families and having single parents impacted similarly on all QOL domains. Family structure had a greater impact on QOL among non-European immigrants because they less often had intact families (46% *vs*. 57% among European immigrants and 64% among French adolescents). Insufficient family income also impacted on all QOL domains, but it was more common among non-European immigrants (32% *vs*. 26% among European immigrants and 17% among French adolescents).

This study further reveals that unhealthy behaviors and violence sustained by adolescents contributed little to poor physical health, psychological health, social relationships, and living environment in addition to socioeconomic factors. In fact, tobacco, cannabis and hard drug use and sexual abuse were much more common among non-European immigrants, while tobacco, cannabis and hard drug use was also, but to a lesser degree, more common among European immigrants than among French adolescents. Overweight similarly affected both European and non-European immigrants. Unhealthy behaviors and violence sustained were differently associated with QOL domains among European and non-European immigrants. Indeed, the present study points out that sexual abuse, and to a lesser degree tobacco and hard drug use, were related to all QOL domains. Cannabis use was related to poor physical health and psychological health only. Overweight was associated with poor physical health, psychological health, and living environment but not with poor social relationships. Therefore, despite the complex association patterns of QOL domains with socioeconomic factors and with unhealthy behaviors and violence sustained by adolescents, parts of these two sets of covariates overlapped for European and for non-European immigrants. Prevention to improve adolescents’ QOL may thus be focused on unhealthy behaviors and violence sustained among immigrants with socioeconomic difficulties. Our study further reports that the relationships between socioeconomic difficulties and unhealthy behaviors and violence sustained greatly differed between French adolescents and immigrants. Therefore prevention should consider the specificities of these groups. 

Our study reveals that, for all the study population, girls were more subject than boys to both poor physical and poor psychological health (gaORs 1.70 and 1.98, respectively). Further analysis shows that these excess risks greatly changed (to 2.00, *p* = 0.02, 95% CI 1.11–3.58 and to 1.51 (non-significant), respectively) with further adjustment for nationality. Interestingly, we found that further adjustment for socioeconomic difficulties clearly reduced the risks (to 1.66, non-significant and to 1.33, non-significant, respectively). This finding indicates that the gender gap for perceived physical and psychological health was partly explained by nationality and more by socioeconomic difficulties. Prevention to reduce the gender gap should thus consider the specific features of boys and girls.

We found that older age was related to poor physical health, psychological health and social relationships (gaOR 1.28, 1.12 and 1.10 per year, respectively) as observed by other studies [13,14]. Further analysis shows that these risks did not change when adjusted for nationality (1.27, 1.11, and 1.08 per year, respectively), moderately changed with further adjustment for socioeconomic difficulties (1.26, 1.08 (non-significant), and 1.06 (non-significant) per year, respectively), and changed more with adjustment for unhealthy behaviors and violence sustained (1.19, 0.98 (non-significant), and 1.05 (non-significant) per year, respectively). This was expected, as these last covariates increased with age. Age could be somewhat considered as an exposure duration from birth but older age means a higher awareness. The increasing risk with age was thus partly explained by socioeconomic difficulties, unhealthy behaviors and violence and it concerned both French and immigrant adolescents. The issues should consequently be evaluated early and addressed to limit their aggravation, especially in late adolescence (16–20 years) and young adulthood [3].

### Limitations and Strengths

This study had some limitations. It was based on self-reported data, but self-administered anonymous questionnaires are widely used and arguably good tools to study adolescent living conditions, mental health, and unhealthy behaviors [3,4,36]. Because of the relatively small number of subjects we did not distinguish between first and second generation immigrants. The study had some strengths. The participation rate was high. The percentage of immigrants is close to that in France [51]. The data collection and weight and height measurements were undertaken by the same trained physician over a short period to avoid inter-observer and seasonal variations. The prevalence of a wide range of health/behavior outcomes, assessed using the same measures, was similar to that of a representative sample of adolescents in France [3,4]. Given the large number of statistical tests performed, type I error may be a concern, but most tests were significant at the 0.001 level, with very high odds ratio estimates. 

## 5. Conclusions

This study on early adolescence in France demonstrates that European immigrants were more subject to poor physical health and poor living environment while non-European immigrants were more subject to all poor physical health, poor psychological health, poor social relationships and poor living environment. It shows that socioeconomic difficulties explained between 20% and 58% of the higher risks, and that parts of the socioeconomic difficulties overlapped with that of unhealthy behaviors and violence sustained by adolescents, while their associations greatly differed between natives and immigrants. These findings of specific risk patterns are important for public policy intended to reduce health-related difficulties, assess need for care, and improve social integration of immigrants. The WHOQOL-BREF appeared to be a good tool, easy to use in a multicultural context to assess and monitor adolescent health-related difficulties. It deserves to be widely used in adolescents. Further studies in other adolescent populations are needed.

## Appendix

**Table A1:** Comparison between the study population and France (ESPAD survey [3,4]): %.

	Study Population (limited to <16 years ^a^) (n = 1,524)	France (ESPAD survey) <16 years
*Number of subjects*	*1,524*	*8,367*
Last-12-month suicide ideation	11.6	9.1
Lifetime suicide attempts	9.6	7.2
Girls	50.1	51.1
Family structure		
Intact	63.2	74.7
Reconstructed	15.0	11.3
Single parent	16.4	11.7
Others	5.4	2.3
Obese (with self-reported data)	10.6	6.9
Last-30-day substance use		
Tobacco	10.7	13.6
Alcohol	34.7	34.6
Cannabis	5.1	5.5
Sleep disorders	32.6	29.0
Asthma	17.2	16.3
Depressive symptoms	13.1	9.8
Sexual abuse	3.4	1.9
Victim of physical/verbal violence (at least once)	53.3	51.5
Involvement in violence (at least once)	59.1	64.7

Note: **^a^** were excluded 35 subjects aged 16 years or over.
